# Hypoxia-Induced Collagen Synthesis of Human Lung Fibroblasts by Activating the Angiotensin System

**DOI:** 10.3390/ijms141224029

**Published:** 2013-12-10

**Authors:** Shan-Shan Liu, Hao-Yan Wang, Jun-Ming Tang, Xiu-Mei Zhou

**Affiliations:** 1 Department of Respiratory Medicine, Beijing Friendship Hospital, Capital Medical University, Beijing 100050, China; E-Mail: liushanshan721cn@126.com; 2 Institute of Clinical Medicine Renmin Hospital, Hubei University of Medicine, Shiyan 442000, Hubei, China; 3 Department of Cardiology, Renmin Hospital, Hubei University of Medicine, Shiyan 442000, Hubei, China; 4 Key Lab of Human Embryonic Stem Cell of Hubei Province, Department of Physiology, Hubei University of Medicine, Shiyan 442000, Hubei, China; 5 Department of Respiratory Medicine, Fengtai Teaching Hospital, Capital Medical University, Beijing 100071, China; E-Mail: zxmei1999@sina.com

**Keywords:** angiotensin II, hypoxic HLF-1, collagen synthesis, nuclear factor κappaB

## Abstract

The exact molecular mechanism that mediates hypoxia-induced pulmonary fibrosis needs to be further clarified. The aim of this study was to explore the effect and underlying mechanism of angiotensin II (Ang II) on collagen synthesis in hypoxic human lung fibroblast (HLF) cells. The HLF-1 cell line was used for *in vitro* studies. Angiotensinogen (AGT), angiotensin converting enzyme (ACE), angiotensin II type 1 receptor (AT1R) and angiotensin II type 2 receptor (AT2R) expression levels in human lung fibroblasts were analysed using real-time polymerase chain reaction (RT-PCR) after hypoxic treatment. Additionally, the collagen type I (Col-I), AT1R and nuclear factor κappaB (NF-κB) protein expression levels were detected using Western blot analysis, and NF-κB nuclear translocation was measured using immunofluorescence localization analysis. Ang II levels in HLF-1 cells were measured with an enzyme-linked immunosorbent assay (ELISA). We found that hypoxia increased Col-I mRNA and protein expression in HLF-1 cells, and this effect could be inhibited by an AT1R or AT2R inhibitor. The levels of NF-κB, RAS components and Ang II production in HLF-1 cells were significantly increased after the hypoxia exposure. Hypoxia or Ang II increased NF-κB-p50 protein expression in HLF-1 cells, and the special effect could be inhibited by telmisartan (TST), an AT1R inhibitor, and partially inhibited by PD123319, an AT2R inhibitor. Importantly, hypoxia-induced NF-κB nuclear translocation could be nearly completely inhibited by an AT1R or AT2R inhibitor. Furthermore pyrrolidine dithiocarbamate (PDTC), a NF-κB blocker, abolished the expression of hypoxia-induced AT1R and Col-I in HLF-1 cells. Our results indicate that Ang II-mediated NF-κB signalling via ATR is involved in hypoxia-induced collagen synthesis in human lung fibroblasts.

## 1. Introduction

Pulmonary fibrosis, particularly idiopathic pulmonary fibrosis (IPF), is a progressive and lethal fibrotic lung disease of unknown aetiology that affects approximately 5 million people worldwide [1]. IPF is associated with progressive lung scarring that subsequently results in the loss of lung function. The incidence of IPF is estimated at 6.8 to 16.3 cases per 100,000 per year in the United States, and there are no currently effective therapeutic strategies. IPF patients have a poor prognosis, with a median survival of 3–5 years [2]. There is a great need for improved knowledge about the disease pathogenesis and for the identification of novel therapeutics.

As the conduit for oxygen uptake, the lungs are affected by hypoxia and hypoxia signalling. Fibroblast proliferation, inflammatory cell infiltration and interstitial thickening can potentially combine with alveolar ventilation defects to increase oxygen consumption and limit oxygen supply to the injured lung, resulting in local tissue hypoxia. Pulmonary tissue oxygen levels are difficult to quantify, but a role for hypoxia in the progression of pulmonary fibrosis has been supported by the observation that increased stabilisation of hypoxia-inducible transcription factor (HIF-1α) has been found in the lung tissues of IPF and cystic fibrosis patients and in mice with bleomycin-induced pulmonary fibrosis [2–4]. Lung fibroblasts have the highest quantity of excreted collagen (mainly I-type collagen), and the proliferation of fibroblasts follow exposure to hypoxia [5]. These results imply that hypoxia could be involved in the development of pulmonary fibrosis. However, little information is available about the molecular mechanisms of hypoxia in pulmonary fibrosis.

The renin-angiotensin system (RAS), an important endocrine system with multiple biological activities, plays an important role in tissue fibrosis [6]. Angiotensin II (Ang II) is a main effector molecule of the RAS and is produced from the substrate angiotensinogen (AGT) through sequential enzymatic cleavage by renin and the angiotensin converting enzyme (ACE). Increases in bronchoalveolar lavage fluid and serum ACE concentrations have been observed in many fibrotic lung diseases, including sarcoidosis, IPF and acute respiratory distress syndrome [7–9]. Ang II generated locally in lung tissues may have autocrine and paracrine actions at the cellular level [10]. Ang II is a potential profibrotic mediator because it induces human lung fibroblasts proliferation and stimulates collagen synthesis [11]. Furthermore, collagen is upregulated in hypoxia-induced lung fibrosis in animal models [12]. However, the molecular mechanisms of hypoxia-induced collagen production in lung fibroblasts remain to be further clarified.

The nuclear factor κappaB (NF-κB) super family of transcription factors has been implicated in the regulation of cell proliferation, cell survival, inflammation, stress, extracellular matrix cross-linking and fibrosis in many cell types, including lung fibroblasts [13]. Previous studies have suggested that NF-κB is involved in Ang II-induced collagen expression in hepatic, kidney and heart tissues [13–15]. Therefore, we hypothesised that Ang II and NF-κB signalling may contribute to collagen synthesis in hypoxic human lung fibroblasts. The aims of this study were to investigate the effects of hypoxia on the components of the RAS, collagen type I (Col-I) protein expression, NF-κB protein expression and nuclear translocation and to further evaluate the relationship between Ang II/ATR and NF-κB signalling during hypoxia exposure in cells.

## 2. Results

### 2.1. Hypoxia Increased Total Collagen and Col-I mRNA and Protein Expression

**Figure 1.**
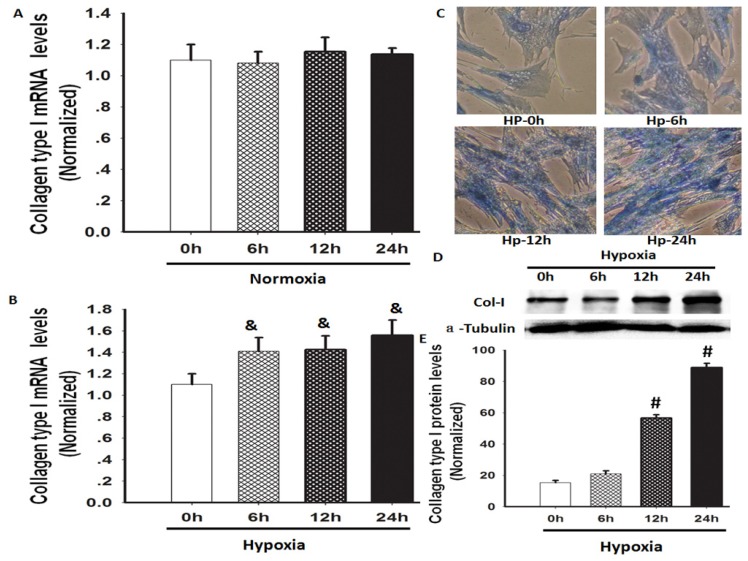
The effects of hypoxia on the total collagen content and Col-I mRNA and protein levels of HLF-1 cells. (**A**,**B**) Col-I mRNA expression was detected using real-time polymerase chain reaction (RT-PCR) in HLF-1 cells cultured for 0, 6, 12 and 24 h under normoxic or hypoxic conditions. The data are presented as means ± standard deviation (SD) (*n* = 3). ^&^*p <* 0.05 *vs.* hypoxia-treated cells at 0 h; (**C**) Masson staining assay was used to observe the changes to the total collagen content in HLF-1 cells cultured under hypoxia for different time periods, as indicated. The total collagen content was increased in a time-dependent fashion after the hypoxic treatment; (**D**) Col-I protein expression was measured using Western blot analysis; and (**E**) Normalisation of the Col-I expression to α-tubulin. The data are presented as means ± SD (*n* = 3). ^#^*p <* 0.05 *vs.* 0 h following hypoxia exposure.

A previous study has shown that hypoxia induces lung fibrosis in an animal model [12]. We investigated whether hypoxia could increase the gene and protein expression of Col-I in human lung fibroblasts (HLF-1). Hypoxia exposure significantly increased the Col-I mRNA and protein expression levels in the cultured HLF-1 cells, but the mRNA expression of Col-I in the cells remained at a similar level under normoxic conditions during the same study period (Figure 1A–E). Total collagen levels are determined by the balance between synthesis and degradation. Herein, we used Masson staining to evaluate the total collagen levels in HLF-1 cells following hypoxia exposure, and we found that the total collagen content was obviously increased. In this study, Col-I expression was measured because it constitutes >65% of total lung collagen in normal human lungs [16].

### 2.2. Hypoxia-Induced AGT and ACE Expression and Ang II Production

**Figure 2.**
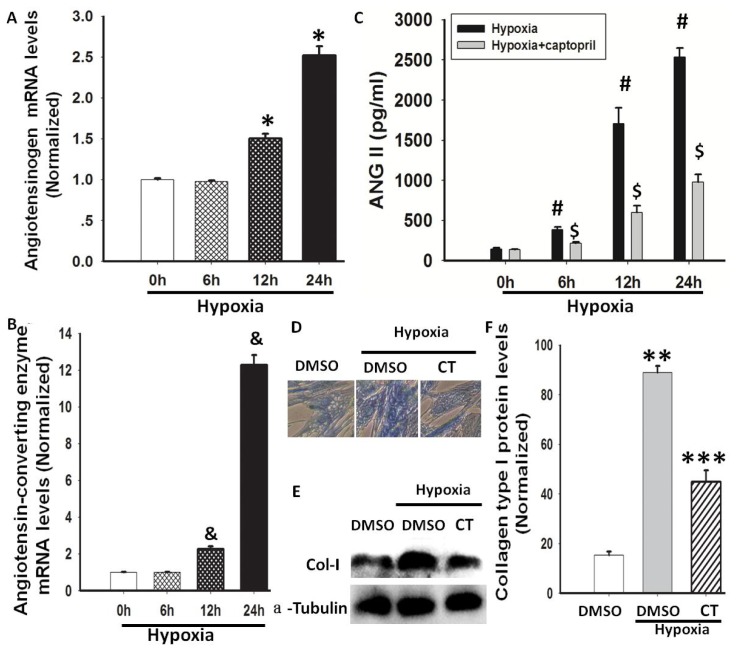
The effects of hypoxia on the AGT and ACE mRNA expression levels and the Ang II level in HLF-1 cells. (**A**) AGT mRNA expression was measured using RT-PCR. The data are presented as means ± SD (*n* = 3). * *p <* 0.05 *vs.* 0 or 6 h following hypoxia exposure; (**B**) The ACE mRNA expression was measured using RT-PCR. **^&^***p <* 0.05 *vs.* 0 or 6 h following hypoxia exposure; (**C**) The effect of hypoxia on the Ang II expression in HLF-1 cells with or without pretreatment with 100 nM captopril for 1 h. The data are presented as means ± SD (*n* = 3). ^#^*p <* 0.05 *vs.* 0 h following hypoxia exposure. ^$^*p <* 0.05 *vs.* different time periods following hypoxia exposure, as indicated; (**D**) A Masson staining assay was used to observe the changes to total collagen content in HLF-1 cells 24 h after the treatment with or without 100 nM captopril (CT) under hypoxia; (**E**) Col-I protein expression was measured using Western blot analysis; and (**F**) Normalization of Col-I expression to α-tubulin. The data are presented as means ± SD (*n* = 3). ** *p <* 0.05 *vs.* DMSO; *** *p <* 0.05 *vs.* 24 h following hypoxia exposure.

The involvement of Ang II, a profibrotic factor, has been confirmed in experimental and clinical lung fibrosis [12,17]; therefore, we investigated whether Ang II was induced in hypoxia-exposed lung fibroblasts. AGT mRNA expression levels in HLF-1 cells and Ang II concentrations in conditioned media from cultured HLF-1 cells were measured. AGT expression was increased in a time-dependent manner, and the values were significantly higher after 24 h of hypoxia exposure (Figure 2A). Ang II is formed from Ang I in a reaction that is catalysed by ACE. Further studies were performed to evaluate whether ACE mediates Ang II synthesis during hypoxia. First, ACE mRNA expression in HLF-1 cells was measured using quantitative RT-PCR, and a significant up-regulation was observed 24 h after hypoxia exposure (Figure 2B). Subsequently, HLF-1 cells were pre-treated with 100 nM captopril for 1 h and then exposed to hypoxic conditions for 24 h. We found that the Ang II levels increased after 24 h of hypoxic treatment and that the ACE blocker captopril inhibited the increase in the Ang II levels (Figure 2C), indicating that ACE contributes to the generation of Ang II in hypoxia-stimulated HLF-1 cells. These results suggested that the combination of AGT and ACE may be responsible for hypoxia-induced Ang II production. At the same time, to observe the effect of hypoxia-induced RAS on collagen expression in HLF-1 cells, we used captopril (100 nM) to block endogenous RAS in HLF-1 cells under hypoxia. We found that captopril inhibited hypoxia-mediated collagen expression in HLF-1 cells (Figure 2D–F). This result suggests that RAS is involved in collagen expression in HLF-1 cells under hypoxic conditions.

### 2.3. Hypoxia Induced both AT1R and AT2R mRNA Expression

**Figure 3.**
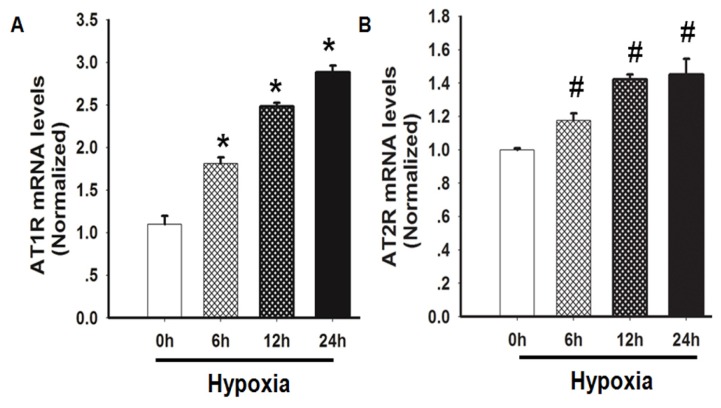
The effects of hypoxia on the AT1R and AT2R mRNA expression levels in HLF-1 cells. HLF-1 cells were treated with hypoxic conditions for up to 24 h. (**A**) AT1R mRNA expression was measured using RT-PCR. The data are presented as means ± SD (*n* = 3). *p < 0.05 *vs.* 0 h following hypoxia exposure; and (**B**) The AT2R mRNA expression was measured using RT-PCR. **^#^***p <* 0.05 *vs.* 0 h following hypoxia exposure.

Both AT1R and AT2R are involved in promoting lung fibrosis via different mechanisms of action [7,12,18]; therefore, the effects of hypoxia on the expression of AT1R and AT2R were also explored in this study. These results showed that AT1R and AT2R exhibited similar responses to hypoxia in HLF-1 cells. The mRNA expression of both AT1R and AT2R increased 6 h after the hypoxic treatment, and the levels were further increased at 12 and 24 h. Furthermore, AT1R mRNA expression was increased to a greater extent than AT2R expression in HLF-1 cells during the same study period (Figure 3A,B).

### 2.4. Hypoxia-Induced Col-I mRNA and Protein Expression via Ang II/ATR Signalling

**Figure 4.**
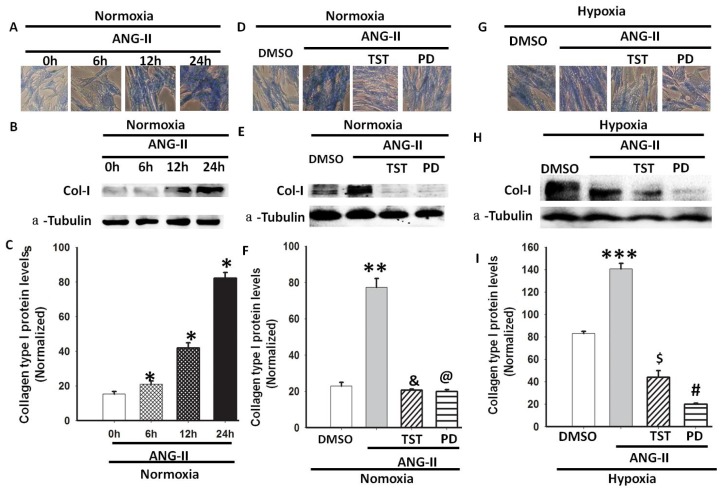
The role of the ATR in Ang II-induced Col-I expression under normoxia and hypoxia. (**A**) A Masson staining assay was used to observe the changes in the total collagen content in HLF-1 cells after treatment with Ang II (1.0 μM) for 0, 6, 12 or 24 h. The total collagen content increased after Ang II treatment in a time-dependent fashion; (**B**) Col-I protein expression was measured using Western blot analysis; (**C**) Normalisation of Col-I expression to α-tubulin. The data are presented as means ± SD (*n* = 3). * *p <* 0.05 *vs.* 0 h following the Ang II exposure; (**D**) A Masson staining assay was used to observe the changes in the total collagen content in HLF-1 cells after Ang II treatment (1.0 μM) for 24 h with the presence or absence of 50 μM TST or 10 μM PD123319 (PD) under normoxic conditions; (**E**) Col-I protein expression was measured using Western blot analysis; (**F**) Normalisation of the Col-I expression to α-tubulin. The data are presented as means ± SD (*n* = 3). ** *p <* 0.05 *vs.* DMSO. **^&^***p <* 0.05 *vs.* Ang II; **^@^***p <* 0.05 *vs.* Ang II; (**G**) A representative picture of the Masson staining in HLF-1 cells treated with Ang II (1.0 μM) for 24 h with the presence or absence of 50 μM TST or 10 μM PD123319 (PD) under hypoxic conditions for 24 h; (**H**) Col-I protein expression was measured using Western blot analysis; and (**I**) Normalisation of Col-I expression to α-tubulin. The data are presented as means ± SD (*n* = 3). *** *p <* 0.05 *vs.* DMSO; **^$^***p <* 0.05 *vs.* Ang II; **^#^***p <* 0.05 *vs.* Ang II.

Ang II has been identified as a profibrotic factor in vascular fibrosis [19]; therefore, we investigated whether Ang II was involved in hypoxia-induced Col-I expression in lung fibroblasts. Firstly, the Col-I mRNA and protein expression levels were measured in HLF-1 cells after Ang II treatment under normoxic conditions. We observed that Col-I expression increased in a time-dependent manner, and the values were significantly higher 24 h after the Ang II treatment (Figure 4A–C). Subsequently, selective Ang II receptor antagonists (telmisartan [TST] for AT1R and PD123319 for AT2R) were used to elucidate the roles of AT1R and AT2R in Ang II-induced collagen production under normoxic conditions. We found that both TST and PD123319 inhibited the Ang II-mediated protein expression of Col-I in HLF-1 cells under normoxic conditions (Figure 4D–F). As shown in Figures 1 and 2, hypoxia not only increased the Col-I expression, but it also induced Ang II synthesis in HLF-1 cells. Further studies were performed to evaluate whether exogenous Ang II promotes Col-I synthesis during hypoxia. TST and PD123319 were also used to elucidate the roles of AT1R and AT2R in exogenous Ang II-induced collagen production under hypoxic conditions. We found that both TST and PD123319 obviously inhibited the protein expression of Col-I in HLF-1 cells incubated with Ang II under hypoxic conditions (Figure 4G–I), and PD123319 inhibited collagen expression to a greater extent than TST (Figure 4I). Taken together, these results suggest that the ATR could play an important role in hypoxia-induced Col-I protein expression.

### 2.5. Hypoxia-Induced NF-κB Expression Involved in the Angiotensin System

**Figure 5.**
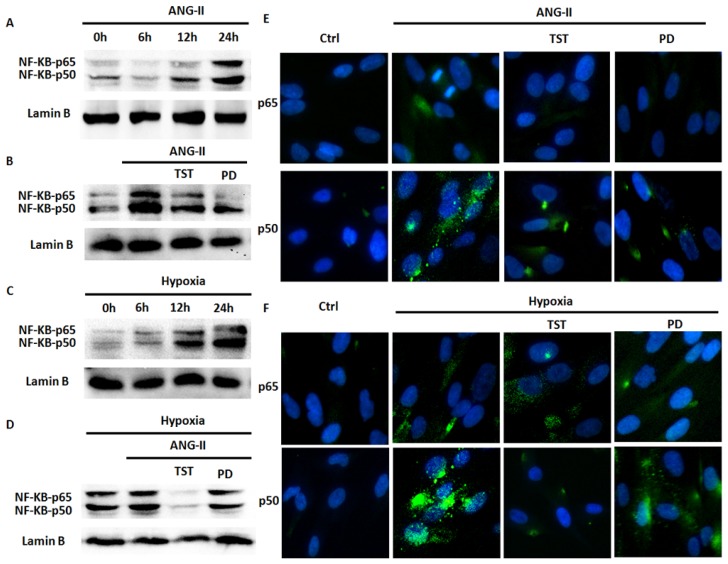
The role of Ang II and ATR in hypoxia-induced NF-κB expression and nuclear translocation. (**A**,**C**) NF-κB protein expression was measured using Western blot analysis. HLF-1 cells were treated with 1.0 μM Ang II for 0, 6, 12 or 24 h; (**B**,**D**) NF-κB protein expression was measured using Western blot analysis. HLF-1 cells were pre-treated with 1.0 μM Ang II for 1 h and pre-incubated with or without 50 μM TST or 10 μM PD123319 (PD) for 1 h and then exposed to normoxia or hypoxia for 24 h; and (**E**,**F**) Immunolocalisation of p65 and p50. HLF-1 cells were pre-treated with 1.0 μM Ang II for 1 h and pre-incubated with or without 50 μM TST or 10 μM PD123319 (PD) for 1 h and then exposed to normoxia or hypoxia for 24 h, after which an immunofluorescence analysis of NF-κB nuclear translocation was performed with fluorescence microscopy.

The NF-κB transcription factor family is involved in controlling multiple aspects of homeostasis, including the functional inflammatory system, immune responses, the cell cycle and cell death in response to various cellular stresses, such as Ang II [20]. Therefore, we investigated whether Ang II is involved in hypoxia-induced NF-κB expression in lung fibroblasts. Firstly, a Western blot analysis was performed to examine the effect of Ang II or hypoxia on NF-κB expression in HLF-1 cells, and we observed Ang II or hypoxia-induced NF-κB (mainly p50) protein expression in a time-dependent manner, which reached peak values 24 h after hypoxia exposure (Figure 5A,C). Subsequently, to determine the relationship between Ang II and hypoxia in NF-κB protein expression in HLF-1 cells, the AT1R selective antagonist TST and AT2R selective antagonist PD123319 were used to elucidate the roles of AT1R and AT2R in hypoxia-induced NF-κB expression. We found that TST could decrease the protein expression of NF-κB in HLF-1 cells mediated by Ang II or hypoxia exposure (Figure 5B–D). Both TST and PD123319 partially inhibited the NF-κB expression mediated by Ang II under normoxic conditions. However, hypoxia-induced NF-κB expression could be completely inhibited in HLF-1 cells after treatment with TST. PD123319 only partially inhibited the protein expression. Finally, confocal immunofluorescence analyses were performed to examine the effect of hypoxia on NF-κB nuclear translocation in HLF-1 cells. We found that Ang II significantly increased the nuclear translocation of p50 in HLF-1 cells after 24 h of normoxia or hypoxia exposure, and hypoxic cells showed a stronger response to the stimulants than normoxic cells (Figure 5E,F). Furthermore, NF-κB expression could be almost completely abolished by TST and partially inhibited by PD123319 after treatment with Ang II under normoxic or hypoxic conditions. Additionally, TST and PD123319 were found to have similar effects on NF-κB nuclear translocation in lung fibroblasts incubated with Ang II under normoxic or hypoxic conditions. These results suggested that the angiotensin system could play an important role in hypoxia-induced NF-κB expression and nuclear translocation in HLF-1 cells.

### 2.6. NF-κB Is Involved in Hypoxia-Induced Col-I Protein Expression Mediated by Ang II/AT1R Signalling

**Figure 6.**
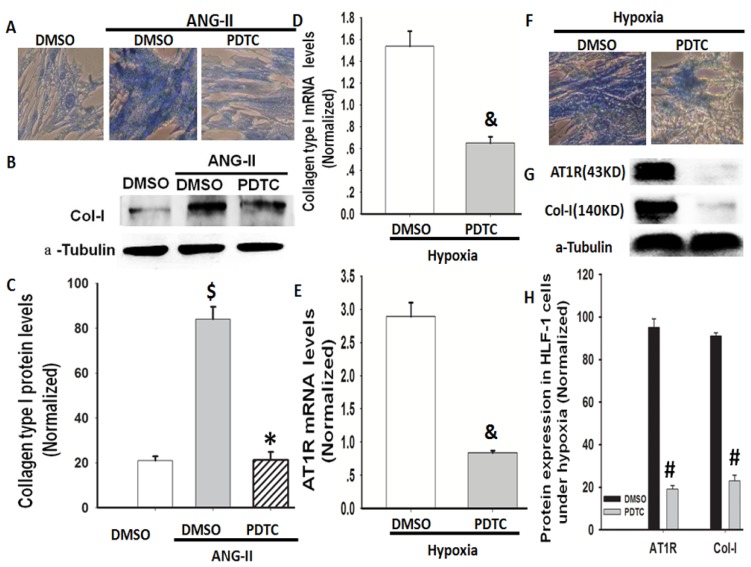
The role of NF-κB in hypoxia-induced Col-I expression under Ang II and hypoxic conditions. (**A**) A representative picture of the Masson staining in HLF-1 cells after the 1.0 μM Ang II treatment with or without PDTC (100 μM) for 24 h; (**B**) The Col-I protein expression level was measured using Western blot analysis; (**C**) Normalisation of the Col-I expression to α-tubulin. The data are presented as means ± SD (*n* = 3). ^$^*p <* 0.05 *vs.* DMSO; * *p <* 0.05 *vs.* Ang II; (**D**,**E**) The Col-I and AT1R mRNA expression levels were measured using RT-PCR. ^&^*p <* 0.05 *vs.* 24 h following hypoxia exposure; (**F**) A representative picture of the Masson staining in HLF-1 cells cultured under hypoxic conditions with or without PDTC (100 μM) for 24 h; (**G**) The Col-I protein expression was measured using Western blot analysis; and (**H**) Normalisation of Col-I expression to α-tubulin. The data are presented as means ± SD (*n* = 3). ^#^*p <* 0.05 *vs.* DMSO.

NF-κB is linked to AT1R activation [21], and NF-κB inhibition ameliorates the Ang II-induced inflammatory damage in rats [22]. Therefore, we investigated whether NF-κB is involved in AT1R and Col-I protein expression under hypoxic conditions in lung fibroblasts. Pyrrolidine dithiocarbamate (PDTC), a selective NF-κB inhibitor, was used to elucidate the role of NF-κB in hypoxia-induced Col-I and AT1R protein expression in HLF-1 cells. First, we found that PDTC decreased Ang II-induced Col-I protein expression in cultured HLF-1 cells under normoxic conditions (Figure 6A–C). Additionally, we found that PDTC abolished not only the hypoxia-induced gene and protein expression of Col-I in the cells but also inhibited AT1R protein expression (Figure 6D–H). The combined data from Figures 4–6 show that NF-κB is involved in hypoxia-induced Col-I protein expression mediated by Ang II/AT1R signalling.

## 3. Discussion

Pulmonary fibrosis is a progressive disease characterised by aberrant repair that results in remodelling and destroying of the normal architecture of the lung tissue, which is associated with persistent or intermittent hypoxia (either globally or regionally) within confined areas of the lung [2]. However, the exact molecular mechanism that mediates hypoxia-induced pulmonary fibrosis still needs to be elucidated. Herein, we used a human lung fibroblast cell line (HLF-1 cells) cultured under hypoxic conditions to imitate the changes in human lung fibroblasts during hypoxia-induced pulmonary fibrosis. Our results show that hypoxia could directly activate the RAS to induce Col-I protein expression mediated by NF-κB signalling, which in turn activated AT1R expression in HLF-1 cells.

Collagen is the major extracellular matrix component of the lungs and is crucial for maintaining normal lung structure and function. Type I collagens are the most abundant collagen subtypes in normal human lungs [23,24]. In this study, Col-I expression was detected and the amplitude of the Col-I expression increased in parallel with the total soluble collagen in hypoxia-exposed HLF-1 cells (Figure 1). Therefore, hypoxia-induced collagen production may be primarily caused by an increase in collagen synthesis rather than a decrease in collagen degradation.

The RAS is a key mediator involved in the pathogenesis of tissue remodelling. Ang II is usually produced by the action of renin on angiotensinogen to form Ang I, which is further cleaved by ACE to generate Ang II [16,25]. Ang II promotes human lung fibroblasts proliferation *in vitro* and stimulates collagen synthesis by activating AT1R [11,26]. Recent studies have shown that the ACE-Ang II-AT1R system serves as a positive feedback loop and fosters lung fibroblast proliferation under hypoxic conditions [27,28]. In this study, we further examined whether hypoxia increased the expression of Col-I, mediated by the ACE-Ang II-AT1R system, and found that hypoxia induced the mRNA expression of AGT and ACE in HLF-1 cells and increased Ang II levels. Meanwhile the ACE inhibitor captopril, AT1R blocker TST and AT2R blocker PD123319 were used to evaluate the role of hypoxia in Ang II-mediated collagen expression in HLF-1 cells. Although we found that TST or PD123319 could inhibit hypoxia-induced Col-I protein expression. The ACE inhibitor reduced Ang II content by approximately 60%—similar to the reduction in Col-I expression. Regarding the ACE and receptor antagonist response, chymase, a non-ACE dependent Ang II-forming serine proteinase could be involved in Col-I expression [29]. Furthermore, the involvement of chymase has been implicated in the fibrotic response (lung fibrosis) to tissue injuries. In addition to its direct action, chymase indirectly promoted the fibrotic response by generating Ang II from Ang I [30,31]. These findings demonstrated that Ang II-AT1R/AT2R was involved in hypoxia-induced collagen expression in HLF-1 cells.

Previous studies have shown that Ang II plays an important role in long-term tissue regulation or organ remodelling through activating NF-κB signalling [32–34]. Additionally, Ang II induced gene transcription related to tissue fibrosis through cell-type-dependent effects on NF-κB signalling [33–38]. The present study showed that Ang II induced the expression of NF-κB and nuclear translocation in HLF-1 cells. Meanwhile, these phenomena were also seen in hypoxia-treated HLF-1 cells. Consistent with the activation of NF-κB, the increased expression of Col-I was simultaneously observed in HLF-1 cells under hypoxic conditions. NF-κB is of particular interest because it is an important mediator of the resynthesis of the Ang II precursor AGT [32], and the activation of AT1R [21]. Herein, hypoxia not only induced NF-κB (mainly p50) protein expression but also resulted in nuclear translocation of NF-κB in HLF-1 cells, which was mediated by Ang II. These effects were abolished by treatment with the AT1R blocker TST and the AT2R blocker PD123319. Furthermore, the activation of NF-κB in turn induced the protein expression of AT1R and Col-I in HLF-1 cells under hypoxic conditions. The specific effect of NF-κB on AT1R and Col-I expression was further demonstrated with PDTC, a selective NF-κB inhibitor. In the present study, we did not detect an effect of PDTC on AT2R expression in these cells. However, previous studies have shown that AT2R could also stimulate collagen synthesis in vessel fibrosis, lung fibrosis, heart fibrosis and other tissues [39,40]. Therefore, we speculate that the interplay of Ang II and NF-κB signalling could play a key role in hypoxia-induced collagen expression in lung fibroblasts. Taken together, hypoxia, RAS and NF-κB likely participate in an integrated system involved in the regulation of lung fibrosis during hypoxemia.

## 4. Materials and Methods

### 4.1. Materials and Reagent

The ACE inhibitor captopril (C4042), AT1R inhibitor TST (T8949), AT2R inhibitor PD123319 (P186), and mouse anti-human α-tubulin (T-6074) antibody were purchased from Sigma-Aldrich (St. Louis, MO, USA). The NF-κB inhibitor PDTC was supplied by Beyotime Institute of Biotechnology (Hangzhou, China). Goat anti-human type I collagen (sc-25974), rabbit anti-human AT1R (sc-1173), rabbit anti-human NF-κB-p65 (sc-109), rabbit anti-human NF-κB-p50 (sc-114), goat anti-human Lamin B Antibody (C-20) (sc-6216) donkey anti-goat IgG-HRP (sc-2304), donkey anti-rabbit IgG-HRP (sc-2305), goat anti-rabbit antibody (sc-372) and goat anti-mouse IgG-HRP (sc-2302) antibodies were purchased from Santa Cruz Biotechnology Inc. (Dallas, TX, USA). Alexa Fluor^®^ 488 Goat Anti-Rabbit IgG (H + L) Antibody (A11008) was purchased from Life Technologies Corporation (Invitrogen, Grand Island, NY, USA).

### 4.2. Cell Culture

HLF-1 cells (human lung fibroblasts; Cell Resource Center of Shanghai Institutes for Biological Sciences, Chinese Academy of Sciences, Shanghai, China) were grown in Dulbecco’s modified Eagle’s medium (DMEM, Gibco, Grand Island, NY, USA), supplemented with 1% penicillin streptomycin and 10% foetal bovine serum (Hyclone, FBS). For each experiment, the cells were grown to confluence and then serum-starved in DMEM containing 0.2% FBS for 24 h. The medium was replaced with FBS-free DMEM under hypoxic conditions. The hypoxic conditions were achieved by placing the cells in sealed chambers filled with 93% N_2_, 5% CO_2_ and 2% O_2_ at 37 °C for the indicated time (STEMCELL Technologies Inc., Vancouver, BC, Canada). The oxygen concentration was checked at the beginning and end of the exposure period by an oxygen analyser. Control cells were kept in normoxic conditions (21% O_2_–5% CO_2_) at 37 °C. In this study, Ang II (1.0 μM, Sigma-Aldrich, St. Louis, MO, USA), TST (50 μM, Sigma-Aldrich, St. Louis, MO, USA). and PD123319 (10 μM, Sigma-Aldrich, St. Louis, MO, USA) were added 1 or 2 h before the hypoxia treatment. The culture medium was replaced every 2 to 3 days until confluence was achieved. The cells were digested with 0.25% trypsin and subcultured. The cells were used for experiments between passages 3 and 6.

### 4.3. RT-PCR Analysis

Total RNA was extracted using a TRIzol kit (Invitrogen, Grand Island, NY, USA). RNA was reverse transcribed in a final volume of 20 μL using 0.5 μg of oligo dT and 200 U Superscript III RT (Invitrogen, Grand Island, NY, USA) for 30 min at 50 °C, followed by 2 min at 94 °C to inactivate the reverse transcriptase. For RT-PCR, the amplification was carried out in a total volume of 25 μL containing 0.5 μM of each primer, 4 mM MgCl_2_ and 12.5 μL of Light Cycler. FastStart DNA Master SYBR green I (Roche Molecular Systems, Indianapolis, IN, USA) and 10 μL of 1:20-diluted cDNA. PCR reactions were prepared in duplicate and heated to 95 °C for 10 min followed by 35 cycles of denaturation at 95 °C for 30 s, annealing at 62 °C for 30 s and extension at 72 °C for 40 s. Standard curves (cycle threshold values *vs.* template concentration) were prepared for each target gene and for the endogenous reference (β-actin) in each sample. The quantification of the unknown samples was performed using the LightCycler Relative Quantification Software version 3.3 (Roche Molecular Systems, Indianapolis, IN, USA). The sequences of the primers are listed in Table 1.

### 4.4. Western Blot Analysis

The nuclear and cytoplasmic protein extracts were prepared according to standard procedures (Beyotime Institute of Biotechnology, Hangzhou, Zhejiang, China) with a protease inhibitor cocktail (Hoffmann-La Roche Ltd, Basel, Switzerland). The protein concentrations were determined using the BCA assay (Pierce, Rockford, IL, USA). Aliquots of protein lysates were separated on sodium dodecyl sulphate (SDS)-10% polyacrylamide gels, transferred onto polyvinylidene fluoride (PVDF) membranes (Millipore, Bedford, MA, USA), blocked with 5% blotting grade milk (Bio-Rad, Philadelphia, PA, USA) in Tris-buffered saline with Tween (TBST, 20 mM Tris-HCl (pH 7.6), 137 mM NaCl, 1% Tween 20) and then probed with the indicated primary antibodies against collagen type I (1:500), AT1R (1:500), NF-κB-p65 (1:500), p50 (1:500), Lamin B (1:500) or α-tubulin (1:5000) at 4 °C overnight. The membrane was washed 3 times with TBST (15 min each time) and then incubated with horseradish peroxidase (HRP)-conjugated secondary antibodies (anti-goat, anti-rabbit IgG and anti-mouse IgG, Santa Cruz Biotechnology, Inc., Santa Cruz, CA, USA, 1:2000). The protein expression was visualised with an enhanced chemiluminescence reaction (Amersham Pharmacia Biotech, Piscataway, NJ, USA) and measured using densitometry. For the Western blot analysis, the densitometry unit of the protein expression in the control cells was assigned as 100% after being normalised to α-tubulin [41].

### 4.5. Ang II Enzyme Immunoassay

The Ang II concentrations in conditioned medium samples were assayed with the commercially available enzyme immunoassay-based colorimetric kit (Raybiotech, Inc., Norcross, GA, USA) [42].

### 4.6. Collagen Assay

The cells were fixed with 4% paraformaldehyde for 15 min and washed 3 times with PBS (5 min each). Then, the cells were stained with a Masson staining kit (Maixin Biotechnology Co., Fuzhou, Fujian, China) [43].

### 4.7. Immunofluorescence Analysis

HLF-1 cells were fixed with 100 μL of 4% formaldehyde in phosphate-buffered saline for 20 min at room temperature, permeabilised with 100 μL of 0.1% Triton X-100 (Maixin Biotechnology Co., Fuzhou, Fujian, China) in phosphate-buffered saline for 5 min at room temperature and then washed twice with 300 μL of 0.1 M Tris-HCl buffer, pH 7.8. To block nonspecific antigenic sites, the wells were incubated for 20 min with 100 μL of 5% goat serum in 0.1 M phosphate buffer (pH 7.8) at room temperature. After washing 2 times in a 0.1 M Tris wash buffer, the cells were incubated overnight at 4 °C with 100 μL of rabbit anti-p50 and anti-p65 NF-κB antibody (Santa Cruz Biotechnology, Inc., Santa Cruz, CA, USA) diluted 1:200 in 0.1 M phosphate buffer, pH 7.8, and 0.1% bovine serum albumin (fraction V; Sigma-Aldrich, St. Louis, MO, USA). The plates were washed 3 times in Tris wash buffer and incubated 30 min at room temperature with 100 μL of goat anti-rabbit–Alexa Fluor 488 (Life Technologies Corporation, Invitrogen, Grand Island, NY, USA, Molecular Probes, 1:100). After several washes with PBS containing 0.1% Triton X-100, the samples were mounted with Vectashield Hardset media containing DAPI (Vector Laboratories, Burlingame, CA, USA), and the images were acquired using a Nikon TiE2000 microscope (Tokyo, Japan).

### 4.8. Statistical Analysis

All data are expressed as means ± SD. A one-way ANOVA was used to compare the variance among several groups and followed by Tukey post hoc test. The significance of the between-group difference was analysed using an unpaired *t*-test, and *p <* 0.05 was considered statistically significant.

## 5. Conclusions

Ang II-mediated NF-κB signalling via ATR is involved in hypoxia-induced collagen synthesis in human lung fibroblasts.

## Figures and Tables

**Table 1.** Gene-specific primers designed for Col-I, AGT, ACE, AT1R, AT2R and β-actin.

Target name	Primer name	Sequence (5′-3′)	Amplicon length
Col-I	Collagen type I	CTTCACCTACAGCGTCACTG GGATGGAGGGAGTTTACAGG	194
AGT	Angiotensinogen	CACCTCGTCATCCACAATGAGA GATGTCTTGGCCTGAATTGG	107
ACE	Angiotensin-converting enzyme	CGACGAGCATGACATCAACT TCTCCTTGGTGATGCTTCCAT	122
AT1R	Angiotensin II receptor type 1	ATCCACCAAGAAGCCTGCAC TGAAGTGCTGCAGAGGAATG	112
AT2R	Angiotensin II receptor type 2	CCTCGCTGTGGCTGATTTACTCCTT TTGCACATCACAGGTCCAA	109
β-actin		GTCCACCGCAAATGCTTCTA TGCTGTCACCTTCACCGTTC	190
